# Effectiveness of the Prospective Prescription Review System in Reducing Irrational Prescriptions at a Tertiary Specialty Hospital: Retrospective Cohort Study

**DOI:** 10.2196/87017

**Published:** 2026-08-27

**Authors:** Fengmin Tang, Min Zhang, Jianwen Shen, Jingchao Yan, Taomin Huang

**Affiliations:** 1 Department of Pharmacy, Eye & ENT Hospital, Fudan University, 83 Fenyang Road, Shanghai, 200031, China, 86 21-64377134

**Keywords:** clinical decision support systems, irrational prescriptions, pharmacists, ophthalmology, otolaryngology, China

## Abstract

**Background:**

The prospective prescription review system can improve prescription rationality, but its effectiveness in high-volume specialty care settings is not well established.

**Objective:**

We aimed to evaluate the effectiveness of the prospective prescription review system in reducing irrational prescriptions and to analyze factors associated with successful interception.

**Methods:**

This retrospective cohort study analyzed all outpatient and emergency prescriptions issued between January 1 and December 31, 2024, at an eye, ear, nose, and throat tertiary hospital in Shanghai, China. Among 2,559,342 prescriptions, 123,914 (4.84%) flagged as irrational by the prospective prescription review system were included.

**Results:**

The overall interception success rate was 32.99% (40,877/123,914). The prescription rationality rate increased from 95.23% (2,476,305/2,600,219) to 96.79% (2,477,144/2,559,342) after excluding intercepted prescriptions (*P*<.001). Higher review levels were strongly associated with higher interception success rates: 0.99% (517/52,146) for reminder, 54.2% (37,039/68,341) for warning, and 96.91% (3321/3427) for mandatory (*P*<.001). Revised prescriptions had a higher interception success rate than prescriptions without revision (35,008/50,418, 69.44% vs 5869/73,496, 7.99%; *P*<.001). Prescriptions without documented reasons showed a higher interception success rate than those with documented reasons (40,086/98,070, 40.87% vs 791/25,844, 3.06%; *P*<.001). Physician acceptance of pharmacist disapproval was associated with a successful interception rate of 85.63% (137/160), compared with 3.33% (2/60) when the disapproval was rejected (*P*<.001). Multivariable logistic regression showed that inappropriate dosage or frequency (odds ratio [OR] 3.11, 95% CI 2.99-3.24) and inappropriate prescription quantity (OR 2.09, 95% CI 2-2.18) were most likely to be intercepted, whereas inappropriate indications (OR 0.04, 95% CI 0.03-0.04) and radiation oncology (OR 0.01, 95% CI 0.01-0.02; *P*<.001) were more likely to be issued. Pharmacist-led rule revision for mometasone furoate nasal spray led to a 1000-fold increase in identified irrational prescriptions (from 2-4 per month to 3022 in May). The interrupted time series analysis showed that the April revision was associated with an immediate and statistically significant increase in the monthly overall interception success rate (intervention coefficient = 0.15; *P =*.005).

**Conclusions:**

The prospective prescription review system substantially reduced irrational prescriptions and improved prescription rationality. Higher review levels were associated with higher interception rates, and physicians’ attitudes also played a critical role. Paired-organ dose standardization emerged as an important consideration in ophthalmology and otolaryngology practice. Collaboration between the prospective prescription review system and pharmacists is essential for optimizing prescription review outcomes.

## Introduction

Computer technology is transforming medical practice by enhancing the quality and efficiency of health care delivery. Electronic prescribing systems, now widely implemented globally, offer significant benefits to stakeholders across the health care system when used effectively [1]. A critical component of these systems is the computerized clinical decision support system (CDSS), which has been shown to improve practitioner performance and clinical practice [2,3]. CDSSs have been reported worldwide to reduce medication error rates [4,5], enhance drug-dosing performance [6], deliver instant information on drug interactions during prescribing and dispensing [7], support antimicrobial stewardship [8], and provide other clinical benefits.

Since 2018, Chinese regulations have mandated that pharmacists be the primary responsible parties for prescription review [9]. The prospective prescription review system has demonstrated effectiveness in promoting rational drug use, reducing costs, and improving clinical outcomes in various clinical settings [10-16]. After implementation of the prospective prescription review system, the number of unreasonable medical orders for inpatients significantly decreased from 540,000 in 2022 to 79,514 in 2024, and the physician modification rate increased from 8.59% to 31.86% [16]. In surgical patients, the length of stay in hospital decreased, with similar readmission rates within 30 days after discharge [11]. In pediatric outpatient settings, the medication rationalization rate increased from 92% to 95.7%, and outpatient medication costs per capita decreased by 3.2% [15]. The alert rates for drug-drug interactions and combined medication errors in obstetrics and gynecology increased 26.1-fold and 26.54-fold, respectively, after implementation of the clinical rules [14]. However, the existing evidence is largely derived from inpatient settings, general medical or surgical departments, obstetric-gynecological, and pediatric care. Nevertheless, detailed analyses of the characteristics and interception of irrational prescriptions, particularly within specialized clinical settings such as ophthalmology and otolaryngology, remain limited. Although previous studies in China have reported improvements in overall prescription rationality, no study, to our knowledge, has specifically detailed the performance and impact of the prospective prescription review system in a high-volume specialty hospital focused on eye, ear, nose, and throat disorders.

To address this evidence gap, we conducted a retrospective cohort analysis of all irrational outpatient prescriptions intercepted by the prospective prescription review system at Eye & ENT Hospital of Fudan University (FDEENT), a leading specialty hospital in China. This study aimed to evaluate the system’s effectiveness in reducing irrational prescriptions, analyze the characteristics of these prescriptions, and elucidate the collaborative roles of the prospective prescription review system and pharmacists in enhancing prescription rationality.

## Methods

### Study Design, Setting, Participants, and Data Collection

We conducted a retrospective analysis of all irrational outpatient prescriptions evaluated by the prospective prescription review system at the FDEENT hospital between January 1, 2024, and December 31, 2024. Data were extracted from prescriptions that the prospective prescription review system had flagged as irrational (N=142,739). After excluding 627 (0.44%) test records and 18,198 (12.75%) duplicate records, 123,914 (86.81%) records were included in the final analysis. As the system automatically recorded all parameters, there were no missing values in the data.

### Ethical Considerations

This study was approved by the ethics committee of the FDEENT in 2025 (2025010). It was conducted in accordance with the Declaration of Helsinki, and the need for informed consent was waived due to the retrospective nature of the study and the use of anonymized data. All patient data were fully anonymized before analysis, with no personally identifiable information accessed by the researchers. No human participants were directly recruited, interviewed, or compensated. No identifiable participant images were included in the manuscript or multimedia appendices.

### The Prospective Prescription Review System

**Figure 1.**
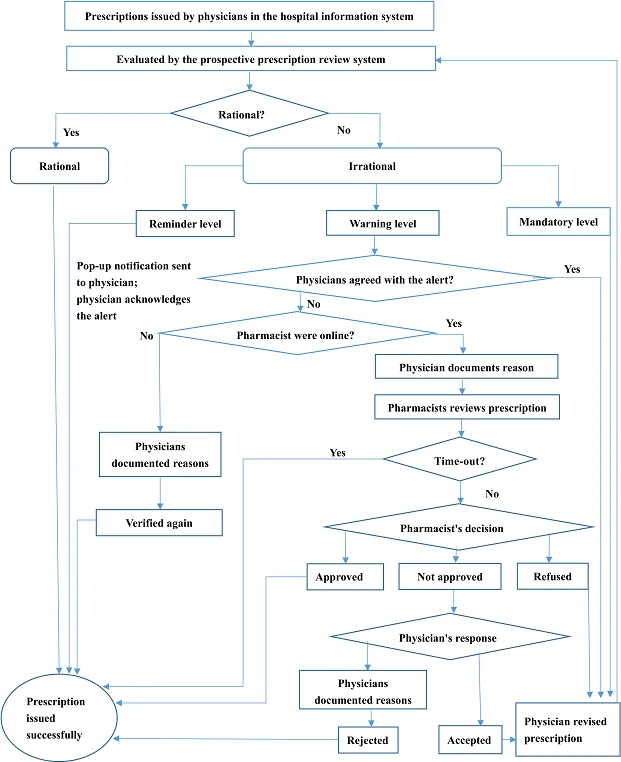
Workflow of the prospective prescription review system.

The prospective prescription review system was developed by Beijing Puhua Health Technology Co Ltd as a rule-based prescription review system. The rule set was derived from national drug package inserts, clinical guidelines, expert consensus statements, and historical prescription data. The system adopted a working model in which it conducted automated preliminary checks, and irrational prescriptions were handled according to the following designated review level:

- Initial evaluation by the prospective prescription review system: after physicians issued prescriptions in the hospital information system (HIS), the prescriptions were first evaluated by the prospective prescription review system according to the established rules. If the prescription’s contents complied with the rules, it was marked as “rational,” successfully issued, and forwarded to the pricing and dispensing stages. If not, it was marked as “irrational” and subjected to further review.
- Categorization of irrational prescriptions: irrational prescriptions were categorized into 3 review levels (ie, reminder level, warning level, and mandatory level). At the reminder level, alerts were sent to physicians for notification only, after which the prescriptions could be issued without revision. At the warning level, if physicians agreed with the alerts, they were required to revise the prescriptions; if they disagreed, they had to document their reasons. At the mandatory level, physicians had to revise the prescriptions; otherwise, the prescriptions could not be issued.
- Pharmacist involvement in irrational prescriptions at the warning level: when pharmacists were not online, physicians had to revise the prescriptions or document their reasons before the prescriptions could be issued. When pharmacists were online, prescriptions with documented reasons were sent to them for review. If pharmacists approved the prescriptions or did not respond within 30 seconds, the prescriptions were issued. If pharmacists refused, physicians had to revise the prescriptions and resubmit them for review.
- Physicians’ response: if physicians accepted the pharmacist’s decision, they had to revise the prescription and resubmit it for review; if physicians rejected the pharmacist’s decision, they had to document their reasons, and the prescription could be issued. The workflow of the prospective prescription review system is illustrated in Figure 1.

### Statistical Analysis

The outcome for irrational prescriptions was “not issued” vs “issued.” The primary measure was the interception success rate, defined as the percentage of prescriptions that were not issued after review. For multivariable adjustment, we included baseline covariates (age, sex, visit type, department, and irrational item) and excluded process-related variables (review level, prescription revision, reason documentation, pharmacist review time-outs, pharmacists’ review decision, and physicians’ responses to disapproval) to avoid tautology.

All analyses were performed using R (version 4.5.1; R Foundation for Statistical Computing). Baseline comparisons used *t* tests and chi-square tests (or Fisher exact tests). Multivariable logistic regression with stepwise selection (both directions, based on the Akaike information criterion) was used to analyze factors associated with “not issued” prescriptions, with odds ratios (ORs) and 95% CIs reported. Multicollinearity was assessed using the variance inflation factor, with values less than 5 indicating acceptable levels. Model discrimination was evaluated using the area under the receiver operating characteristic curve with a 95% CI, and calibration was assessed using the Hosmer-Lemeshow goodness-of-fit test (*g*=10). An interrupted time series analysis was performed using segmented linear regression with Huber-White robust SEs to estimate the preintervention trend, immediate level change, and postintervention slope change, from which monthly intervention effects and 95% CIs were derived via counterfactual predictions. All tests were 2-sided, with *P*<.05 considered statistically significant.

## Results

### Characteristics of Irrational Prescriptions

During the study period from January 2024 to December 2024, a total of 2,559,342 prescriptions were issued at the FDEENT Hospital. Of these, 123,914 (4.84%) were identified as irrational by the prospective prescription review system, with an overall interception success rate of 32.99% (n=40,877 prescriptions intercepted before dispensing). The prescription rationality rate increased from 95.23% (2,476,305/2,600,219) at baseline to 96.79% (2,477,144/2,559,342) after excluding intercepted prescriptions, representing an absolute increase of 1.55 percentage points (*P*<.001).

As shown in Table 1, the mean age of the “issued” group was 44.05 (SD 21.8) years, compared with 28.58 (SD 25.38) years in the “not issued” group (*P*<.001). Significant differences between groups were observed for all variables (all *P*<.001). The “not issued” group had a higher proportion of male patients (22,042/40,877, 53.92% vs 41,068/83,037, 49.46%), emergency visits (2279/40,877, 5.58% vs 3467/83,037, 4.18%), and otorhinolaryngology department visits (31,984/40,877, 78.24% vs 41,619/83,037, 50.12%). Regarding irrational items, the “not issued” group showed higher proportions of inappropriate prescription quantity (9487/40,877, 23.21% vs 12,009/83,037, 14.46%) and inappropriate dosage or frequency (20,828/40,877, 50.95% vs 9906/83,037, 11.93%), but lower proportions of inappropriate indications (693/40,877, 1.7% vs 39,981/83,037, 48.15%).

This retrospective analysis included 123,914 prescriptions identified by the prospective prescription review system at a tertiary ophthalmology and otolaryngology specialty hospital in Shanghai, China, between January 2024 and December 2024. The interception success rate was defined as the percentage of prescriptions not issued after review.

**Table 1.** Characteristics and interception success rates of irrational prescriptions identified by the prospective prescription review system (N=123,914).

Variables	Total (N=123,914)	“Issued” group (n=83,037)	“Not issued” group (n=40,877)	Interception success rate (%)	*P* value
Age (years), mean (SD)	38.94 (24.17)	44.05 (21.8)	28.58 (25.38)	32.99	<.001^a^
Sex, n (%)					<.001^b^
Male	63,110 (50.93)	41,068 (49.46)	22,042 (53.92)	34.93	
Female	60,804 (49.07)	41,969 (50.54)	18,835 (46.08)	30.98	
Visit type, n (%)					<.001^b^
Emergency	5746 (4.64)	3467 (4.18)	2279 (5.58)	39.66	
Outpatient	118,168 (95.36)	79,570 (95.82)	38,598 (94.42)	32.66	
Department, n (%)					<.001^b^
Stomatology	1048 (0.84)	810 (0.98)	238 (0.58)	22.71	
Plastic and reconstructive surgery	1295 (1.04)	951 (1.14)	344 (0.84)	26.56	
Radiation oncology	2685 (2.17)	2639 (3.18)	46 (0.11)	1.71	
Ophthalmology	45,283 (36.54)	37,018 (44.58)	8265 (20.22)	18.25	
Otorhinolaryngology	73,603 (59.4)	41,619 (50.12)	31,984 (78.24)	43.45	
Irrational item, n (%)					<.001^b^
Others	21,194 (17.1)	14,045 (16.91)	7149 (17.49)	33.73	
Duplicate medication	9816 (7.92)	7096 (8.54)	2720 (6.65)	27.71	
Inappropriate prescription quantity	21,496 (17.35)	12,009 (14.46)	9487 (23.21)	44.13	
Inappropriate dosage or frequency	30,734 (24.8)	9906 (11.93)	20,828 (50.95)	67.77	
Inappropriate indications	40,674 (32.82)	39,981 (48.15)	693 (1.69)	1.7	

a *P* value calculated using the *t* test.

b *P* value calculated using the chi-square test.

### Characteristics of Irrational Prescription Review Process and Interception Success Rates

Table 2 presents the characteristics of the irrational prescription review process and interception success rates. Review level was significantly associated with interception success rate (*P*<.001): the success rate was 0.99% (517/52,146) for the reminder level, 54.2% (37,039/68,341) for the warning level, and 96.91% (3321/3427) for the mandatory level. Revised prescriptions had a higher interception success rate than prescriptions without revision (35,008/50,418, 69.44% vs 5869/73,496, 7.99%; *P*<.001). Prescriptions without documented reasons showed a higher interception success rate than those with documented reasons (40,086/98,070, 40.87% vs 791/25,844, 3.06%; *P*<.001). Pharmacist review was rare overall (1,384/123,914, 1.12%) and had a lower interception success rate than prescriptions without pharmacist review (243/1384, 17.56% vs 40,634/122,530,33.16%; *P*<.001).

**Table 2.** Prescription review process variables and interception success rates of review level, prescription revision, documentation of reasons, and pharmacist review status (N=123,914).

Variables	“Issued” group (n=83,037), n (%)	“Not issued” group (n=40,877), n (%)	Interception success rate (%)	*P* value^a^
Review level				<.001
Reminder	51,629 (62.18)	517 (1.26)	0.99	
Warning	31,302 (37.7)	37,039 (90.61)	54.2	
Mandatory	106 (0.13)	3321 (8.12)	96.91	
Prescription revision				<.001
No	67,627 (81.44)	5869 (14.36)	7.99	
Yes	15,410 (18.56)	35,008 (85.64)	69.44	
Documentation of reasons				<.001
No	57,984 (69.83)	40,086 (98.06)	40.87	
Yes	25,053 (30.17)	791 (1.94)	3.06	
Pharmacist review status				<.001
No	81,896 (98.62)	40,634 (99.41)	33.16	
Yes	1141 (1.37)	243 (0.59)	17.56	

a *P* values were calculated using the chi-square test.

### Pharmacist Review and Interception Outcomes

As shown in Table 3, among 1384 pharmacist-reviewed prescriptions, 243 (17.56%) were not issued. Prescriptions with time-outs had a higher interception success rate than those without time-outs (25.08% vs 15.52%; *P*<.001). Approved prescriptions had a 3.34% success rate (29/868), whereas disapproved prescriptions had a 63.18% success rate (139/220; *P*<.001). Among physicians who accepted disapproval, 85.63% (137/160) of prescriptions were not issued, compared with 3.33% (2/60) among those who rejected disapproval (*P*<.001).

**Table 3.** Pharmacists’ review process variables and interception success rates of review time-outs, review decisions, and physicians’ responses to disapproval (N=1384).

Variables	“Issued” group, n (%)	“Not issued” group, n (%)	Interception success rate (%)	*P* value
Pharmacists’ review time-outs^a^			17.56	<.001^b^
No	920 (80.63)	169 (69.55)	15.52	
Yes	221 (19.37)	74 (30.45)	25.08	
Pharmacists’ review decision^c^			15.52	<.001^d^
Approved	839 (91.2)	29 (17.16)	3.34	
Disapproved	81 (8.8)	139 (82.25)	63.18	
Refused	0 (0)	1 (0.59)	100	
Physicians’ responses to disapproval^e^			63.18	<.001^b^
Accepted	23 (28.4)	137 (98.56)	85.63	
Rejected	58 (71.6)	2 (1.44)	3.33	

a Pharmacists’ review time-out analysis included 1141 issued and 243 not issued prescriptions.

b *P* value calculated using the chi-square test.

c Pharmacists’ review decision analysis included 920 issued and 169 not issued prescriptions.

d *P* value calculated by the Fisher exact test.

e Physicians’ responses to disapproval analysis included 81 issued and 139 not issued prescriptions.

### Factors Associated With Prescription Interception

**Figure 2.**
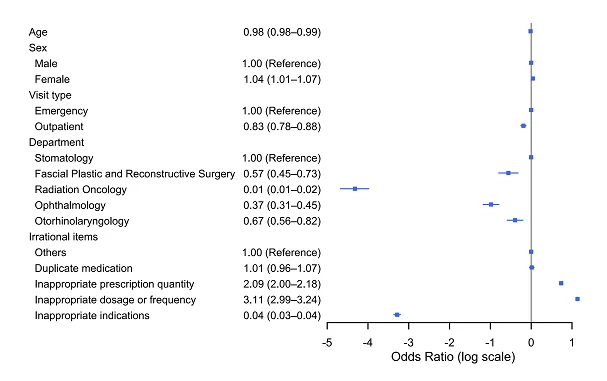
Forest plot of multivariable analysis of factors associated with interception success rate (N=123,914).

The multivariable logistic regression showed that inappropriate dosage or frequency (OR 3.11, 95% CI 2.99-3.24; *P*<.001) and inappropriate prescription quantity (OR 2.09, 95% CI 2-2.18; *P*<.001) were the factors most strongly associated with prescriptions being not issued among irrational prescriptions. Conversely, inappropriate indications (OR 0.04, 95% CI 0.03-0.04; *P*<.001) and radiation oncology (OR 0.01, 95% CI 0.01-0.02; *P*<.001) were more likely to be issued, suggesting that these prescriptions were less likely to be intercepted by the system. Details are shown in Figure 2.

No multicollinearity was detected (all adjusted generalized variance inflation factor values <1.05). The model showed good discrimination (area under the receiver operating characteristic curve 0.852, 95% CI 0.85-0.854). However, the Hosmer-Lemeshow test indicated poor calibration (*χ*^2^_8_=1215.84; *P*<.001).

### Characteristics of Inappropriate Dosage of Mometasone Furoate Nasal Spray

To investigate why irrational prescriptions were more frequently not issued to male patients and younger patients, we analyzed all irrational prescriptions. The most common reason was an inappropriate dosage of mometasone furoate nasal spray (17,729/123,914, 14.3%), of which 15,113 (85.24%) were not issued. This subgroup was predominantly male (11,114/17,729, 62.69%) and significantly younger than the group without inappropriate mometasone furoate dosage (mean age 6.68, SD 2.68 vs 44.33, SD 21.85 years; both *P*<.001). From January 2024 to March 2024, prior to the rule revision, monthly prescriptions with inappropriate mometasone furoate dosage numbered 2 to 4. On April 15, 2024, pharmacists revised the dosage rules for children aged 3 to 11 years. Following this change, identified irrational prescriptions surged to 17.49% (1799/10,284) in April and 26.69% (3022/11,323) in May, compared with the previous rate of 0.02% (2/10,552), marking an increase of up to 1000-fold.

**Figure 3.**
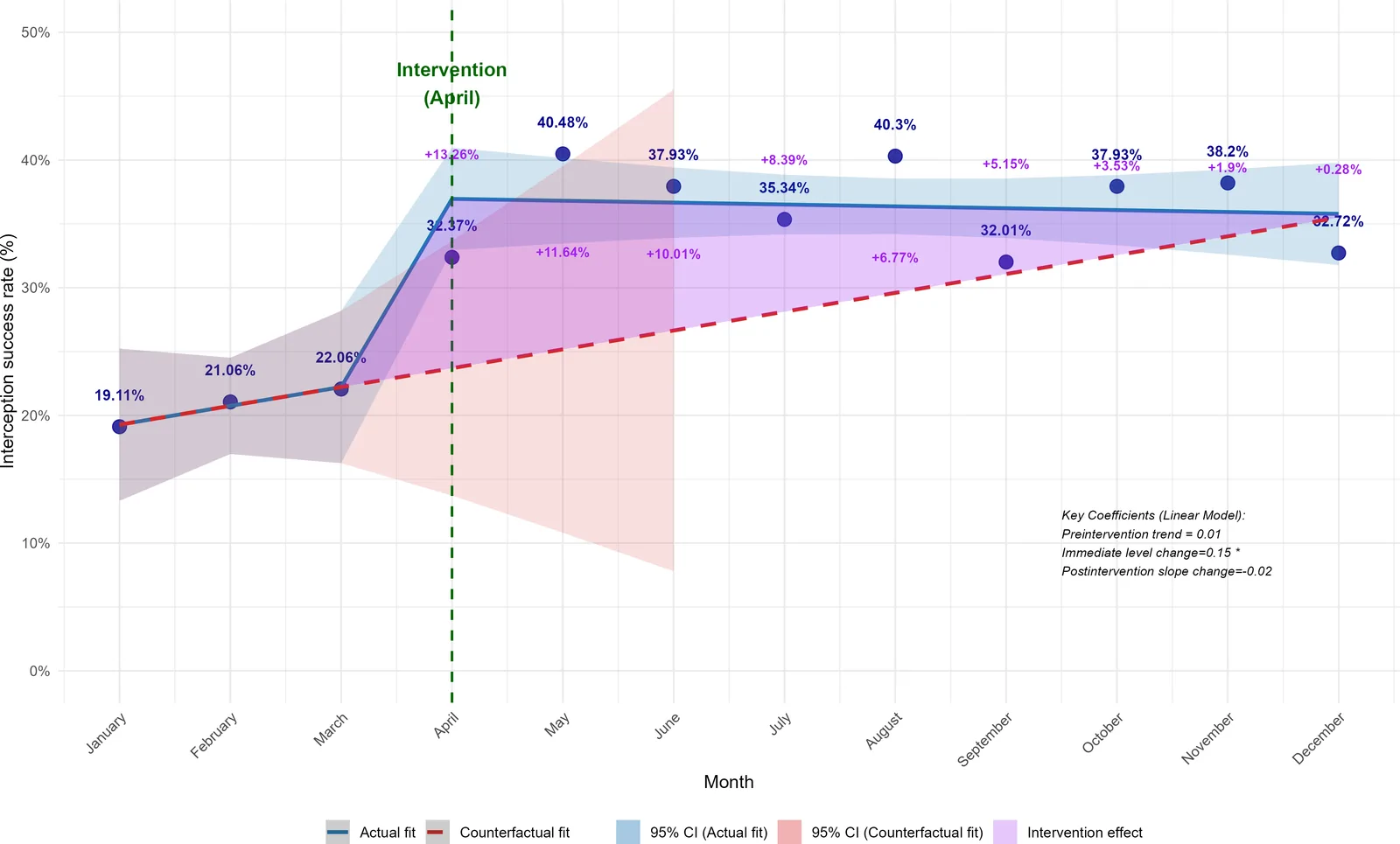
Interrupted time series analysis of the effect of the April 2024 system revision on the monthly interception success rate (N=123,914).

An interrupted time series analysis (Figure 3) showed that the monthly overall interception success rate increased gradually from 19.11% (1918/10,036) in January to 22.06% (1971/8934) in March during the preintervention period. Following the April revision, an immediate and statistically significant level increase to 32.37% (3263/10,079) was observed (intervention coefficient= 0.15; *P*=.005). The success rate peaked at 40.48% (4513/11,150) in May and subsequently fluctuated between 32.01% (2819/8807) and 40.3% (4547/11,283) throughout the remainder of the postintervention period. By December, the success rate had decreased to 32.72% (4258/13,015), approaching the counterfactual predicted level (intervention effect 0.28%).

## Discussion

### Principal Findings

In this large retrospective cohort study involving more than 2.5 million outpatient prescriptions from a tertiary specialty hospital, the prospective prescription review system was associated with an incremental yet statistically significant improvement in prescribing rationality, leading to the interception of 40,877 irrational prescriptions. The absolute increase of 1.52% is particularly notable given that the baseline rationality rate was already high. To our knowledge, this is one of the few studies to evaluate a prospective prescription review system in a high-volume ophthalmology and otolaryngology specialty hospital, where prescribing challenges are compounded by paired-organ (eg, bilateral eye, ear, or nose) dosing considerations. Our findings are consistent with previous evaluations of the prospective prescription review system, which have similarly demonstrated substantial reductions in irrational prescribing and improvements in prescribing rationality following implementation [15-18]. These findings highlight the complementary role of the prospective prescription review system in supporting pharmacist-led prescription review.

### Clinical Implications of Irrational Prescription Patterns

The 3 most frequent categories of irrational prescriptions were inappropriate indications, inappropriate dosage or frequency, and inappropriate prescription quantity, accounting for nearly three-quarters of all irrational prescriptions. These findings are generally consistent with previous studies evaluating prospective prescription review systems in general hospitals [10,19,20]. Previous studies reported a higher prevalence of allergic rhinitis in boys than in girls among children [21-23]. Consequently, irrational prescriptions that were not issued were more likely to be for male patients and younger patients.

In ophthalmology and otolaryngology, distinguishing between unilateral and bilateral involvement constitutes a critical clinical observational parameter. This is evident in sudden sensorineural hearing loss, where the bilateral or unilateral distinction guides counseling and management [24], and in cataract surgery, where failure to mark the operative side can result in a high error rate, a risk that extends to surgeries on other paired organs [25]. Our findings highlight a distinctive challenge in ophthalmology and otolaryngology prescription review: for medications applied to paired organs—whether eyes, ears, or nostrils—HISs and the prospective prescription review system must explicitly define whether dose fields represent unilateral or bilateral administration, with standardized definitions to prevent systematic errors. The initial failure to detect these errors stemmed from a misalignment between the HIS default dosage settings and the prospective prescription review system review rules. According to the package insert of the mometasone furoate nasal spray, the recommended regimen is 1 spray per nostril for children aged 3 to 11 years (total of 2 sprays) and 2 sprays per nostril for adults (total of 4 sprays). The HIS had defaulted the “single-dose” field to 2, interpreted as sprays per nostril (consistent with the adult regimen), whereas the prospective prescription review system originally treated this value as the total for both nostrils combined. Consequently, when physicians prescribed the spray for children aged 3 to 11 years and retained the default value of 2, the prospective prescription review system interpreted this as 2 sprays total—which is appropriate for children—whereas the actual administration corresponded to 2 sprays per nostril (4 sprays total), representing a clear overdose, yet no alert was triggered regarding the inappropriate dosage. This cross-system discrepancy in field definition could be identified and reconciled only by pharmacists possessing both medication dosing knowledge and system-configuration expertise. This finding demonstrates that the prospective prescription review system cannot replace pharmacists’ professional judgment.

### Higher Review Levels Associated With Higher Interception Rates

Higher review levels were associated with substantially higher interception rates. Mandatory-level alerts achieved near-complete interception (3321/3427, 96.91%), warning-level alerts achieved an interception rate of 54.2% (37,039/68,341), and reminder-level alerts were largely disregarded (517/52,146, 0.99% interception success rate). Inappropriate dosage or frequency and inappropriate prescription quantity were the types most likely to be intercepted, as they were mostly set at the warning review level. Inappropriate indications and radiation oncology were more likely to be issued because their review levels were downgraded to reminders.

CDSSs have been shown to result in alert fatigue and high override rates [26,27]. We recommend that pharmacists adjust review levels accordingly: contraindications should be set to the mandatory level, inappropriate dosage or frequency may be managed at the warning level, and low-risk categories (eg, inappropriate indications) may be appropriately assigned to the reminder level to minimize unnecessary interruption of clinical workflow.

### The Importance of Physicians’ Acceptance in Interception Outcomes

Physicians’ attitudes substantially influenced interception outcomes, consistent with prior research confirming that review systems can improve prescribing practices, yet their effectiveness depends on physician acceptance [28,29].

Physician acceptance of system alerts was associated with a substantially higher interception success rate than nonacceptance. Interception success was markedly higher for revised prescriptions than for those without revision (35,008/50,418, 69.44% vs 5869/73,496, 7.99%; *P*<.001). Similarly, physician acceptance of pharmacist disapproval yielded an interception success rate of 85.63% (137/160) vs 3.33% (2/60) when physicians rejected the disapproval (*P*<.001). In contrast, prescriptions with documented reasons for retaining the original prescription had a lower interception success rate (791/25,844, 3.06%) than those without documented reasons (40,086/98,070, 40.87%). Future strategies should involve frontline clinicians in rule development and alert design to enhance system acceptability.

### CDSS From Domestic and International Perspectives

CDSS tools integrated with electronic health records (EHRs) are increasingly used to guide clinical practice across diverse care teams, including physicians, advanced practice professionals, nurses, technicians, social workers, care managers, and allied health professionals [30]. Major EHR platforms such as Epic (Epic Systems Corp) and Cerner (Oracle Health) are widely adopted in the United States, Europe, and other regions, including the Middle East [31-33]. However, evidence suggests that the Epic CDSS tools demonstrate only modest real-world performance with high intersite heterogeneity, underscoring the need for local validation prior to deployment [34]. Similarly, the Cerner PowerChart offers limited out-of-the-box functionalities for optimization efforts, requiring substantial institutional information technology resources [35]. In China, the medical information technology infrastructure is well established [36], and due to health care policy and data localization requirements, numerous domestic EHR systems are in use. The prospective prescription review system is a CDSS designed to support pharmacists by providing prospective evaluation of prescriptions for both inpatients and outpatients prior to dispensing.

Internationally, the World Health Organization and the International Pharmaceutical Federation have published a series of documents and tool kits emphasizing medication safety as a core priority during the medication process [37]. The International Pharmaceutical Federation highlights that medication review and medication use review services are indispensable, promoting patient safety by avoiding medication errors and reducing drug-related harm, while improving treatment outcomes through enhanced drug effectiveness, safety, patient adherence, and engagement. Pharmacists are expected to play a leading role in these services. The prospective prescription review system aligns with these international goals by supporting pharmacist-led medication review.

### Collaboration Between the Prospective Prescription Review System and Pharmacists

Although the prospective prescription review system automatically reviewed all prescriptions in real time and ensured efficient screening in this high-volume outpatient setting, it could not fully replace pharmacist expertise. Pharmacist burnout is driven by long working hours, high patient volumes, and excessive workloads [38], with elevated workload also increasing the risk of dispensing potential drug-drug interactions [39]. Although CDSSs can offer substantial time savings for clinical pharmacists [40]—a critical advantage under staffing constraints—our findings reaffirm that pharmacists remain essential for system optimization. In our study, the prospective prescription review system alone detected almost no inappropriate pediatric dosing cases for mometasone furoate nasal spray until pharmacists identified the root cause (inconsistent interpretation of the dosage field) and updated the review rules. This approximately 1000-fold increase in detection following pharmacist-led rule refinement highlights the necessity of pharmacist involvement. These findings indicate that the system cannot replace pharmacists but can serve as a complementary tool, requiring the integration of pharmacist expertise with system capabilities.

### Limitations and Future Work

This study has several limitations. First, the Hosmer-Lemeshow test was significant; however, this statistic is overly sensitive in large samples. Second, the single-center retrospective design at a tertiary specialty hospital limits the generalizability of our findings. Third, we relied on prescribing rationality as a surrogate end point without assessing downstream clinical outcomes (eg, adverse drug events and hospitalizations) associated with intercepted prescriptions. Finally, without a clinical gold standard, we could not assess unflagged prescriptions or calculate false-negative rates or true specificity; thus, the system’s true accuracy remains unverified. Therefore, multicenter prospective studies incorporating clinical outcome assessment are needed to validate the wider applicability of these results and to establish the patient-level impact of the prospective prescription review system.

The rapid advancement of AI, particularly in machine learning and deep learning, has further expanded the potential of CDSSs to improve diagnostic accuracy, optimize workflow, reduce medical errors, and empower patients [41-43]. Future work should focus on enhancing AI capabilities through machine learning and deep learning to better recognize irrational prescriptions and improve prescription rationality.

### Conclusions

The prospective prescription review system substantially reduced irrational prescriptions and improved prescription rationality. Higher review levels were associated with higher interception rates. Physicians’ attitudes also played a critical role in intercepting irrational prescriptions. Paired-organ dose (both eyes, both ears, or both nostrils) standardization is an important issue in ophthalmology and otolaryngology practice.

These findings have broader implications for the design of CDSSs and the prospective prescription review system. The successful implementation of the prospective prescription review system in a high-volume ophthalmology and otolaryngology specialty hospital suggests its potential scalability to other specialty settings, contingent on specialty-specific rule customization. The stark contrast between the near-complete interception rate of mandatory alerts (3321/3427, 96.91%) and the negligible impact of reminder-level alerts (517/52,146, 0.99%) not only reflects physicians’ alert fatigue—manifested as the widespread neglect of low-level alerts—but also validates the value of pharmacist-optimized tiered alerting. The fact that paired-organ dosing errors were detected only through pharmacist-led rule refinement reinforces that the prospective prescription review system serves as a complement to, rather than a replacement for, pharmacists. With the continued advancement of machine learning, its integration with pharmacist expertise represents a promising direction for further improving prescribing rationality in specialty care.

## Supplementary material

10.2196/87017 Checklist 1 STROBE checklist for cohort studies.

## Abbreviations

CDSS: clinical decision support system
EHR: electronic health record
FDEENT: Eye & ENT Hospital of Fudan University
HIS: hospital information system
OR: odds ratio
